# Exploring Family Typologies and Health Outcomes in a Dutch Primary Care Population of Children Living in Urban Cities in the Netherlands: A Latent Class Analysis

**DOI:** 10.3390/ijerph22101474

**Published:** 2025-09-24

**Authors:** Samantha F. F. Groenestein, Matty R. Crone, Evelien M. Dubbeldeman, Stijntje Lottman, Jessica C. Kiefte-de Jong, Jet Bussemaker, Suzan van der Pas

**Affiliations:** 1 Department of Public Health and Primary Care, Leiden University Medical Center, 2300 RC Leiden, The Netherlands; e.m.dubbeldeman@lumc.nl (E.M.D.); stijntjelottman@hotmail.com (S.L.); j.c.kiefte@lumc.nl (J.C.K.-d.J.); m.bussemaker@lumc.nl (J.B.); s.van_der_pas@lumc.nl (S.v.d.P.); 2 Department of Health Promotion, Maastricht University, 6200 MD Maastricht, The Netherlands; matty.crone@maastrichtuniversity.nl; 3 Institute of Public Administration, Leiden University, 2511 DP The Hague, The Netherlands; 4 Faculty of Social Work & Applied Psychology, Leiden University of Applied Sciences, 2333 CK Leiden, The Netherlands

**Keywords:** family typologies, latent class analysis, child health, neighborhood livability, early life vulnerability, family demographics

## Abstract

This study examined social and physical environmental exposures, health, and healthcare utilization among children aged 0–12 in urban areas. A population-based cross-sectional design was used, incorporating general practitioners’ data (2018–2019, *n* = 14,547), and societal and environmental data. Latent class analysis identified three distinct classes based on child and family demographics: ‘Dutch-origin two-parent household’ (*n* = 7267), ‘households with diverse countries of origin’ (*n* = 4313), and ‘single-parent household’ (*n* = 2967). Binary and multinomial logistic regression examined associations with environmental factors and child health outcomes. Children from the Dutch-origin class most often had favorable family demographics, neighborhood conditions, and health outcomes. Children from the diverse countries of origin class most often faced adverse neighborhood conditions, had higher rates of physical or somatic health conditions, and higher healthcare costs. Children from the single-parent class more often had less favorable family demographics, a higher likelihood of mental health problems, more frequent general practitioner visits, and were often in contact with youth care. This study highlights how child and family demographics and social and neighborhood conditions impact child health and healthcare utilization. Future approaches should focus on strategies to build and strengthen family and community resilience and adopt family-centered, context-sensitive interventions.

## 1. Introduction

Early social or family adversity can significantly affect an individual’s physical and mental health outcomes throughout their life [1,2]. Although adverse health outcomes may not always manifest before the age of five, early-life risk factors may contribute to early-life health inequalities [3,4,5]. Bronfenbrenner’s socioecological systems theory emphasizes the significant effect of individual, social, and environmental factors on child development. These factors act as either risks or protective elements for health outcomes. As such, for young children, growing up in good health partially depends on the conditions into which they are born [4,6,7].

According to this theory, the immediate environment of a child (microsystem), including their parents, directly interacts in a bidirectional manner with the child [8]. Family circumstances, such as socioeconomic status (SES), can create significant disparities in a child’s development [9,10,11]. Previous literature indicates that children and adolescents from low-SES households have a two- to threefold increased risk of developing mental health problems [12]. One study found that children from households with low perceived SES are more likely to experience diverse mental health issues, such as behavioral, concentration, or emotional problems, compared to those from high perceived SES households [10]. One explanation is heightened family stress related to employment, housing conditions, interpersonal tensions, and health issues [10]. Additionally, family dynamics, such as transitions between two-parent households and one-parent households, are associated with the development of mental health problems in children [13]. 

Children from low-SES households or neighborhoods are more likely to grow up in unhealthy environments, with poor air quality and limited exposure to greenery [14,15,16,17,18]. This physical environment, including safety and social cohesion in the neighborhood, directly influences a child’s behavior. Previous research even suggested a positive association between parents’ perception of neighborhood safety and their child’s health [19]. Additionally, the neighborhood’s infrastructure and facilities could directly influence a child’s health behavior, opportunities for physical activity, and parents’ perception of their child’s health [8,19,20].

Much of the existing literature on social inequality does not account for the complex interactions between social and environmental factors. This study extends the literature by using latent class analysis (LCA) to classify family types using a range of child demographics and family factors, referred to as ‘family demographics’. In addition, by explaining the association of family types with environmental factors and child health, we illustrate the complexity of early-life vulnerability and the implications for interventions. To the best of our knowledge, this is the first study to examine general child and family demographics in a representative child population from urban cities in a Western society using general practitioners’ (GPs) data, generalizable routinely collected data, and the Dutch Livability Index data [21]. 

This study aims to (1) identify family types based on child and family demographics of children aged 0–12 years in two Dutch urban cities, (2) analyze their social and physical environmental exposures, and (3) examine the impact of family types on the health and healthcare utilization of the children.

## 2. Materials and Methods

This population-based cross-sectional study is part of the project ‘Countering syndemic vulnerability: A community resilience approach.’ The project aims to develop an approach for building community resilience in vulnerable neighborhoods in the Netherlands using the syndemic theory.

In this study, data from the Extramural Leiden University Medical Centre Academic Network (ELAN) [22,23] for 2018 and 2019 were used to avoid the influence of the COVID-19 pandemic on health and healthcare utilization. The ELAN contains medical primary care records from inhabitants in The Hague and Leiden regions, including GP-registered disease episodes coded according to the WHO International Classification of Primary Care (ICPC) [22,23,24]. These data were combined with societal data from the System of Social Statistical Datasets (SSD) provided by Statistics Netherlands (SN) and 2018 data from the Dutch Livability Index from the Ministry of the Interior and Kingdom Relations. The SSD includes sociodemographic, socioeconomic, household, and healthcare utilization data [25]. Based on the Livability Index, we formulated five social and physical environmental factors related to the living environment, i.e., the neighborhood, of children [26]. All datasets were anonymized and linked using unique ID numbers. Parental GP and SSD data were correlated with children’s data using a unique parent–child identification number from SN, while the Livability Index data were connected using neighborhood codes.

### 2.1. Study Population

The study population consisted of children who were 0–12 years of age as of 1 January 2018, and registered with a participating GP in The Hague or Leiden regions. In the Netherlands, GPs typically serve patients from their neighborhoods, which ensures that the GP-registered population accurately reflects the local pediatric population (*n* = 95,425 in 2018) [27,28]. The exclusion criteria were death in 2018 or 2019, having been registered with a participating GP for less than 12 months, or the absence of information on parental health or societal data for both parents.

### 2.2. Child and Family Demographics

To examine child and family demographics, we included a variety of variables from the individual, household, and parental dimensions. These variables and their corresponding time periods were selected for their relevance to the Dutch context and their known impact on child health, development, and adversity [4,11,29]. A detailed description of these variables is provided in Table A1.

The individual dimension included the child’s demographics, such as age, gender, country of origin (determined by their birthplace and that of their parents), and social problems (e.g., family issues) as recorded during a GP visit.

The household dimension covered the situation the child lives in, such as family situation (e.g., divorce of parents in the past five years, single-parent household, or household composition), household income, home ownership, and mean neighborhood SES. 

The parental dimension included parental data on the highest achieved educational level; physical, mental, and somatic health conditions; and social problems (e.g., those recorded during a GP visit, crime victimization, criminal detention or suspicion, or having debts).

### 2.3. Social and Physical Environmental Factors

To analyze the association between social and physical environmental factors and types of family demographics, we formulated five factors based on the Livability Index, which influence neighborhood livability [30]:

(1) Physical environment includes data such as the distance to roads and green areas, air quality, and noise pollution.
(2) Housing stock includes data such as residential area, housing vacancy, and overcrowding.
(3) Facilities stock includes proximity data, such as the distance to healthcare, education, and hospitality service providers.
(4) Social cohesion includes aspects such as population density and diversity of life stages.
(5) Nuisance and insecurity, which include violent crimes, vandalism, public disturbances, and experienced nuisance and insecurity [30].

For each livability factor, an outcome of zero corresponds to the Dutch average, i.e., the outcome values represent a deviation from this average. All the social and physical environmental features of each factor, including categorization, are presented in Table A2.

### 2.4. Health Outcomes

To compare and determine the well-being of children across different types of family demographics, we analyzed their health conditions, healthcare utilization, healthcare costs, and being in contact with youth care.

The selection of the health conditions was based on their significant impact on the children’s health and well-being (Table A3) [31,32]. In this study, ‘health conditions’ refer to chronic health conditions or those that have long-lasting effects. We classified these into physical (e.g., asthma and epilepsy), mental (e.g., externalizing behavioral and internalizing disorders), and somatic (psychosomatic complaints, e.g., generalized fatigue/pain and headaches) health conditions.

In the Netherlands, individuals are required to visit a GP for referrals to specialized care and for the prescription of medication, with the exclusion of repeat prescriptions. GP visits are fully covered under the Health Insurance Act, which entails that no additional costs have to be borne by the individual. Symptoms and diagnoses are registered by a GP by using the ICPC, which were used in this study to identify health conditions. The two-year prevalence of conditions was determined by the presence and sum of still-active diagnoses in 2018 or 2019. The children were categorized as having no conditions (no morbidity), one condition (single morbidity), or multiple conditions (multimorbidity). 

Healthcare costs considered in this study include all the expenses covered under the Health Insurance Act in 2018 and 2019, such as those related to the GP and hospital care, with the exclusion of costs related to youth care. Further, there are no deductibles for any care covered by the basic health insurance for children up to 18 years of age. 

Being in contact with (inpatient or outpatient) youth care was analyzed as a dichotomous variable. A ‘yes’ indicated that the child had received one or more types of assistance or care, such as support for mental health problems or parenting issues, provided under the Youth Act and Youth Protection Services from 2018 up to and including 2019. 

### 2.5. Statistical Analysis

The data were prepared using IBM SPSS Statistics Version 25 (IBM, New York, NY, USA), while the statistical analyses were conducted using RStudio (version 4.2.3) (R Core team, Vienna, Austria). Descriptive statistics were employed to describe the child and family demographics of the entire study population, and the environmental factors and health outcomes of the children in each latent class.

### 2.6. Latent Class Analysis

LCA was performed to obtain distinct groups of children (latent classes) based on their child and family demographics. It divides a heterogeneous group into homogeneous subgroups based on patterns in the given variables. Latent classes were assessed based on entropy, with higher values reflecting clearer class separation [33]. Additionally, classes were evaluated for distinctiveness, relevance, theoretical grounding, and interpretability of their results (Table A4). 

### 2.7. Logistic Regression Analyses

Unadjusted multinomial logistic regression analyses were carried out to explore the relationship between each individual social and environmental factor and the latent classes. Binary logistic regression analyses were conducted to assess the associations between belonging to a certain latent class and physical, somatic, and mental health conditions, and being in contact with youth care. In addition, separate unadjusted multinomial logistic regression analyses were conducted to explore the interrelations between the number of health conditions, GP visits, and healthcare costs and the latent classes. All the regression analyses were performed with a significance level of *p* < 0.05. 

## 3. Results

The study population consisted of 14,547 children aged 0–12 years. A majority of them were 0–4 years old, with the age groups being relatively evenly distributed, and almost half of them were of Dutch origin. Most of the children lived in a household that had more than three members, a moderate household income, was owner-occupied, and was located in a low-SES neighborhood. Further, a majority of the parents of the children had a high educational level, while one of the parents suffered from a physical and/or somatic health condition.

### 3.1. LCA Class Interpretation

**Figure 1:**
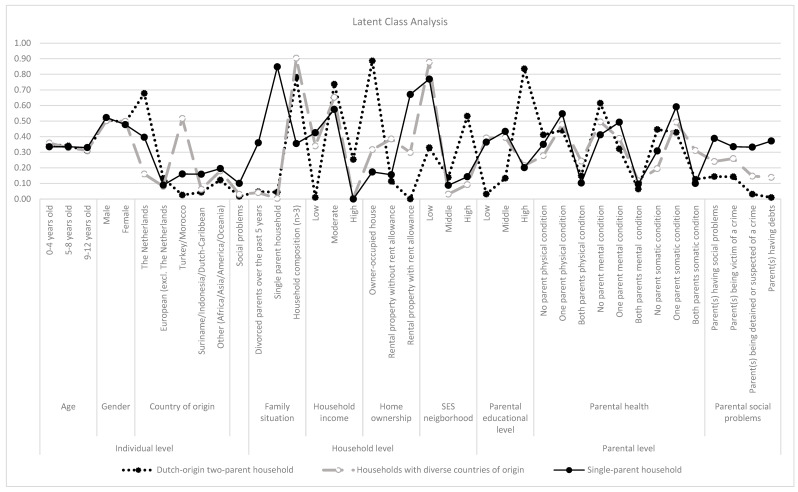
Visual representation of probability prevalence rates of the child and family demographics according to the three classes identified through LCA.

We selected the three-class model as the final model based on its fit indices, as it had the highest entropy value of 0.82 (Appendix A Table A4) Additionally, it provided the most meaningful interpretation, as all the classes exhibited the most diverse patterns across the variables. The class proportions and the probability prevalence of the child and family demographics are detailed in Table 1 and shown in Figure 1. The three identified classes were labeled according to the highest probability of the child and family demographics being prevalent in that class: ‘Dutch-origin two-parent household’, ‘households with diverse countries of origin’, and ‘single-parent household’.

#### 3.1.1. Class 1: ‘Dutch-Origin Two-Parent Household’ (*n* = 7267 [50% of Total Study Population])

Of all the children included in this study, almost half were classified into Class 1, which was predominantly characterized by a high likelihood of children who were born in the Netherlands and whose parents were both born in the Netherlands. The children from this class were more likely to live in an owner-occupied house, belong to families with moderate or high income, reside in a high-SES neighborhood, and live in a household that had more than three members. They were most likely to have highly educated parents, with neither of them suffering from a mental or somatic health condition.

#### 3.1.2. Class 2: ‘Households with Diverse Countries of Origin’ (*n* = 4313 [29.7% of Total Study Population])

Almost 30% of all the included children were classified into Class 2. They were characterized by greater diversity in country of origin, with a relatively higher likelihood of being born in Turkey or Morocco, or having one or both parents born in Turkey or Morocco. The children from this class were more likely to live in households that had more than three members, had a low or a moderate income, and were located in a low-SES neighborhood. Furthermore, one or both of the parents of these children were more likely to have been suffering from physical, mental, or somatic health conditions, to have social problems registered by their GP, and to have been a victim of a crime.

#### 3.1.3. Class 3: ‘Single-Parent Household’ (*n* = 2967 [20.4% of Total Study Population])

The third class had the lowest prevalence, comprising a fifth of the sample population. It was characterized by several prominent adverse family demographics. A significant characteristic was the high likelihood of children living in a single-parent household, with their parents having gotten divorced within the past five years. These children were more likely to be living in a household with low or moderate income and in a rental property with rent allowance located in a low-SES neighborhood. Moreover, the parents of these children were more likely present physical, mental, or somatic health conditions, to have social problems registered by the GP, to have been a victim of a crime, to have a history of being detained by the police or to have been suspected of a crime, and to have financial debts compared to those of the children from Class 1 and 2.

### 3.2. Social and Physical Environment

The results of the unadjusted multinomial logistic regression analyses of the social and physical environmental factors and latent classes revealed significant differences in livability scores (Table 2). 

The children from Class 1 generally scored equal to or above the Dutch average for almost all livability variables (Table A5). In contrast, the children from Class 2 were the least likely to score near the average for physical environment, nuisance and insecurity, social cohesion, and housing stock, showing a stronger tendency toward large negative deviations. However, they were more likely than Class 1 to live in environments with a large positive deviation for the facilities factor. 

The children from Class 3 also tended to show negative deviations from the Dutch average for physical environment, nuisance and insecurity, social cohesion, and housing stock. However, they had slightly better odds than the children from Class 2 of scoring closer to the Dutch averages. Notably, their likelihood of a large positive deviation for the facilities factor was lower than that of the children from Class 2. 

### 3.3. Child Health-Outcomes 

The logistic regression analyses (Table 3) revealed significant differences in child health outcomes across all the classes. The children from Class 1 were most likely to have the best health outcomes. In contrast, the children from both Class 2 and Class 3 exhibited increased health vulnerabilities and higher levels of healthcare utilization (Table A6). 

Children with multiple health conditions and mental health conditions had a higher likelihood of belonging to Class 3. Although the children from Class 2 were the least likely to have a mental health condition, children with physical and somatic health conditions were more likely to belong to Class 2. 

In terms of healthcare utilization, children having high healthcare costs were more likely to belong to Class 2, while those who had greater use of GP services or had contact with youth care were more likely to belong to Class 3.

## 4. Discussion

The aim of this study was to explore clustered child and family demographics of 0–12-year-old children from two Dutch urban cities, The Hague and Leiden, and the associated social and physical environmental exposures, health, and healthcare utilization. Using LCA, we identified three distinct classes: ‘Dutch-origin two-parent household’, ‘households with diverse countries of origin’, and ‘single-parent household’. Each family type was found to possess its own specific characteristics that distinguished it from the others. Differences were also found in terms of social and physical environmental circumstances, and the children in each class presented differences in health outcomes, healthcare utilization, and healthcare costs. These differences highlight potential protective and risk factors for health outcomes. 

The children from Dutch-origin households, the majority of the sample, showed relatively favorable health outcomes despite some reports of mental health conditions. This suggests that the family demographics and environmental factors of these children functioned as protective factors. This included higher parental income and education levels, and living in a high-SES neighborhood with favorable social and physical environmental factors. Such factors are consistently identified within previous studies, which reported these factors as being protective across all areas of child development [7,11,16,17,20].

In contrast, children from households with diverse countries of origin face moderate but significant vulnerabilities. They exhibited greater vulnerability to physical or somatic health conditions and high healthcare expenditure. Overall, this class was characterized by relatively more adverse family demographics, such as lower household income and parents with physical, mental, or somatic health conditions. These findings can be explained by Bronfenbrenner’s ecological systems theory, which states that the material possessions and economic position of a family are vital factors that affect child development and early-life inequalities [34]. This explains why children growing up in low-SES households have unhealthier outcomes compared to those who grow up in middle- or high-SES households [11]. Further, the children in this class more often resided in low-SES neighborhoods with poor social and physical environmental factors, including further distance to green areas, nuisance and insecurity, less social cohesion, and low housing stock. Such environmental factors were linked to physical complaints and stress-related health outcomes in children in previous studies [19,35,36]. The finding of frequent or chronic pain (e.g., pain in their head, body, or dorsal area) in one study aligns with the physical and somatic health conditions, including complaints of pain and headaches, presented by the children from households with diverse countries of origin in our study [35]. One study described the built living environment as a predictor of social interaction, linking health with both stress responses and behaviors, such as physical activity [36]. Therefore, we suggest that the unfavorable social and physical environmental factors in the neighborhoods of children from households with diverse countries of origin negatively influence their health behavior and potentially impact their health outcomes [20].

The children from single-parent households experienced the most pronounced disadvantages. This was, with 20%, the smallest group, but they were most likely to have multiple health conditions, particularly mental health conditions, and had the highest GP visits and contact with youth care. Their adverse family demographics highlight the complex manner in which the setting in which children grow up can influence the development of early mental health problems [37,38,39,40]. Family adversities in this class, including parental financial debts; social problems; a history of criminal detention or suspicion; and a physical, mental, or somatic health condition, were also found in the previous literature and more often present than in two-parent households [39]. The combination of such adverse factors or life events, generally referred to as adverse childhood experiences (ACEs), can negatively affect brain development at a young age, increasing the risk of mental health problems, attention-deficit/hyperactivity disorder (ADHD), tobacco use, and engaging in dangerous behaviors later in life [41,42,43,44,45,46]. ACEs, such as parental divorce, the death of one’s father, the witness of paternal violence toward one’s mother, and an upset male guardian, have been identified as risk factors for household poverty in both childhood and adulthood [45,47]. Single-parent households often resided in low-SES neighborhoods in rental properties with rent allowance. Although neighborhood conditions were better than those of households with diverse countries of origin, these neighborhoods are described in the literature as associated with mental health risks [46,48]. 

The combination of adverse social circumstances, health conditions (mental, physical, and somatic), and unfavorable social and physical environments highlights the importance of a syndemic approach for understanding adverse family outcomes. The syndemic theory posits that the co-occurrence of health conditions that interact within a social and environmental context leads to worse health outcomes than the occurrence of a single health condition in isolation [49]. A previous Dutch study suggested that this syndemic vulnerability may be intergenerational and highlighted the need for interventions to focus on syndemic vulnerability within families [50]. Our findings show the onset of health problems at a young age among children growing up in contexts with unfavorable family demographics and adverse environmental and social factors, placing them at risk for reduced quality of life and health inequalities [7,51,52]. Furthermore, experiencing such adversities during childhood can have long-term health effects throughout one’s life [29,53,54]. 

One approach to breaking the cycle of intergenerational vulnerabilities is the three-generation approach. In this three-generation approach, the focus is on parents (the first generation), their children (the second generation), and the future offspring of these children (the future generation). The key principle is long-term investment to reduce vulnerability among all children to improve health across generations [55]. Elements to reduce vulnerability include policy support for investments in, and accessibility to, education and health services, such as pregnancy-related care visits, and social and community services, and collaboration among all stakeholders [55]. We believe that family and community resilience play a key role in child development and in reducing vulnerability. The building community resilience framework serves as a model for strengthening and building community resilience coalitions for child ACEs. It emphasizes system change and facilitates collaboration among relevant stakeholders who all directly or indirectly impact a child’s health, and overall health of the community [56,57]. In addition, targeted, systemic interventions are needed for families living in disadvantaged neighborhoods and experiencing complex vulnerabilities, such as a low socioeconomic position, parental mental health problems, and single parenthood. Interventions should be family-centered, taking family demographics into account instead of focusing on individual problems or single family members. Additionally, it is important to assess what the family actually needs in order to cope with vulnerability. Therefore, a key element of such interventions should be the joint assessment of family or child care needs through conversations with parents and their children, followed by shared decision-making [58]. A practical example involves social workers operating in schools who provide support to families, collaborate with educational staff, and facilitate connections with relevant stakeholders in the health and social domain [59,60]. 

A significant strength of this study was that we used a combination of societal, social, and physical environmental data, along with registered GP data, which made it possible to include a wide variety of variables over time. By utilizing routinely collected electronic data, we eliminated the potential bias associated with socially desirable responses in self-reported data. Further, in the Netherlands, GPs play a gatekeeping role, as individuals are required to visit a GP for referrals to specialized care and for prescriptions for medication. Although some residents may not be registered with a GP or may limit their visits, we suggest that using GP data reduces the risk of non-participation in the study. Another strength of this research is that LCA is an effective and accurate technique to identify classes and could potentially be superior to other techniques. Lastly, our study sample was representative of children living in urban cities in the Netherlands.

This study has certain limitations that should be addressed. First, we were not able to obtain some information about the family demographics at the parental dimension, as only one parent was registered for some children in either the ELAN or SN. In addition, we did not include information on siblings, living with stepfamilies, or co-parenting arrangements, although these factors may play a vital role in the child’s development and the family’s situation. Second, since we used GP data, it is possible that the number of children having one or more health conditions is higher, as they may not always visit the GP when they have symptoms, and as we included a selection of health conditions. In addition, not all GPs in both cities participated in the ELAN, and families may have a GP outside of the city they live in. In this study we were not able to account for potential implicit bias of GPs, which may have influenced the diagnoses made and treatments provided [61]. However, in the present study we anticipated this limitation in advance and therefore discussed the data with GPs. Nevertheless, it remains a topic that requires attention and training. Lastly, we did not have any data on immigration status, such as how long families live in the Netherlands. Future research should adopt a longitudinal approach to explore syndemic vulnerability in families and its progression over the course of time. In addition, it would be valuable to include information about other (family) members in the household situation. Finally, although there are multiple family-centered methods, future studies should focus on methods involving an intergenerational approach, its key components, and its effectiveness on family outcomes [62].

## 5. Conclusions

Through latent class analysis of family demographics, we identified three distinct classes, each characterized by specific risk and protective factors related to child health outcomes. The differences in health conditions (mental, physical, and somatic) and healthcare utilization between the classes highlight the complexity of unfavorable family demographics associated with adverse social and physical environments. The children from Dutch-origin households showed more protective factors and relatively favorable health outcomes. In contrast, the children from households with diverse countries of origin faced more risk factors combined with physical or somatic health conditions, and higher healthcare expenditure. The more pronounced disadvantages were observed among the children from single-parent households, who were most likely to have adverse health outcomes, particularly mental health conditions, and higher levels of healthcare utilization. 

These findings emphasize that clustered vulnerability factors, such as socioeconomic disadvantage, adverse environmental exposures, unfavorable household situations, and parental health problems, exacerbate the risk of early health inequalities. This study demonstrates that adopting a syndemic framework would be useful for understanding the complex interactions between co-occurring health conditions and social factors in an urban setting. A next step in using a syndemic framework would be to explore whether and how these health, social, and environmental conditions in children interact and might lead to poorer childhood outcomes than expected. Additionally, our findings highlight the importance of an intergenerational perspective, as many of the observed vulnerabilities may be structural inequalities across generations, in need of early and structured interventions.

Overall, our results show the relevance for policy makers and health and social services to work with strategies to build and strengthen family and community resilience. Strategies include policy support and multidomain collaboration, as framed by the building community resilience approach. From a practical perspective, interventions should be long-term and multigenerational, such as the three-generation approach, which aims to improve health across current and future generations. In addition, more attention should be directed to families navigating combinations of unfavorable family demographics and adverse social contextual factors, which can serve as early indicators for adverse health outcomes. Interventions and future approaches should adopt family-centered and context-sensitive strategies that address the family as a whole, rather than focusing on individual problems or single family members. Conversations with parents and their children, facilitated by social workers at schools, and shared decision-making are crucial steps in assessing what a family truly needs to cope with vulnerability. 

## Figures and Tables

**Table 1:** Probability prevalence rates ^1^ of the child and family demographics according to the three classes ^2^ identified through LCA, and distribution of the total study population of children aged 0–12 years.

	Class 1	Class 2	Class 3	Total Study Population
	Dutch-origin two-parent household (n = 7267)	Households with diverse countries of origin (n = 4313)	Single-parent household (n = 2967)	n (%) (n = 14,547)
Estimated class population shares	49.2	29.5	21.3	
Predicted by modal posterior probability	50.0	29.7	20.4	
** Individual level **				
Age ^3^ **				
0–4 years old	** 35.0 **	** 35.9 **	** 33.5 **	5087 (35.0)
5–8 years old	34.1	32.9	33.5	4889 (33.6)
9–12 years old	30.9	31.2	33.0	4571 (31.4)
Gender ^3^ **				
Male	** 50.8 **	** 50.2 **	** 52.3 **	7410 (50.9)
Female	49.2	49.8	47.7	7137 (49.1)
Country of origin ^3^ **				
Netherlands	** 67.8 **	16.0	** 39.6 **	6762 (46.5)
European (other than the Netherlands)	13.3	8.1	8.9	1571 (10.8)
Turkey/Morocco	2.6	** 51.7 **	16.0	2899 (19.9)
Suriname/Indonesia/Dutch-Caribbean	4.3	6.2	15.9	1068 (7.3)
Other(Africa/Asia/America/Oceania)	12.1	18.0	19.5	2247 (15.4)
Social problems ^3^	1.7	3.0	** 10.0 **	561 (3.9)
** Household level **				
Family situation				
Divorced parents over the past 5 years ^6^	4.7	4.1	** 36.1 **	1629 (11.2)
Single parent household ^3^ **	4.4	0.3	** 84.8 **	2961 (20.4)
Household composition (n > 3) ^3^ **	** 78.1 **	** 90.4 **	35.6	10607 (72.9)
Household income ^3^ **				
Low	1.0	34.0	42.5	2852 (19.6)
Moderate	** 73.7 **	** 65.2 **	** 57.5 **	9851 (67.7)
High	25.4	0.7	0	1844 (12.7)
Home ownership ^3^ *				
Owner-occupied house	** 88.6 **	31.7	17.3	8234 (56.6)
Rental property without rent allowance	11.4	** 38.5 **	15.6	2950 (20.3)
Rental property with rent allowance	0	29.8	** 67.1 **	3363 (23.1)
SES neighborhood ^4^ *				
Low	33.0	** 87.7 **	** 76.9 **	8511 (58.5)
Middle	13.8	3.1	8.8	1395 (9.6)
High	** 53.2 **	9.2	14.3	4641 (31.9)
** Parental level **				
Parental educational level ^5^ **				
Low	3.2	39.2	36.5	2770 (19.0)
Middle	13.3	** 39.5 **	** 43.4 **	3634 (25.0)
High	** 83.5 **	21.4	20.1	6763 (46.5)
Missing				1380 (9.5)
Parental health **				
No parent physical condition ^7^	41.1	27.8	35.1	5223 (35.9)
One parent physical condition ^7^	** 44.1 **	** 47.9 **	** 54.7 **	6905 (47.5)
Both parents physical condition ^7^	14.8	24.3	10.2	2419 (16.6)
No parent mental condition ^7^	** 61.5 **	** 49.5 **	41.2	7797 (53.6)
One parent mental condition ^7^	32.2	38.9	** 49.3 **	5499 (37.8)
Both parents mental condition ^7^	6.3	11.7	9.6	1251 (8.6)
No parent somatic condition ^7^	** 44.6 **	19.5	30.9	4983 (34.3)
One parent somatic condition ^7^	42.8	** 49.4 **	** 59.2 **	7012 (48.2)
Both parents somatic condition ^7^	12.7	31.1	10.0	2552 (17.5)
Parental social problems				
Parent(s) having social problems ^7^ **	14.4	** 24.0 **	** 38.9 **	3268 (22.5)
Parent(s) being victim of a crime ^6^	14.3	** 26.0 **	** 33.5 **	3177 (21.8)
Parent(s) being detained or suspected of a crime ^6^	3.1	14.7	** 33.3 **	1886 (13.0)
Parent(s) having debts ^6^ **	0.9	13.9	** 37.2 **	1818 (12.5)

The numbers in bold represent variables with the highest prevalence between the classes (by row). The definitions and details of the variables are provided in Table A1. A chi-squared test was performed on the categorical variables. * *p* < 0.05. ** *p* < 0.001. ^1^ Prevalence is determined by the posterior probability that the social contextual factor is present in the latent class. ^2^ The latent class assignment of individuals was based on the highest posterior probability for each class. ^3^ As registered in the year 2018. ^4^ As registered in the year 2019. ^5^ As registered in the years 2015–2018. ^6^ As registered during the period of 2015–2019. ^7^ Based on the ELAN registration data of parents between 2016 and 2019.

**Table 2:** Multinomial logistic regression results of the defined social and physical environmental factors in 2018 for the latent classes (reference category: Class 1 ‘Dutch-origin two-parent household’).

	Class 2			Class 3		
**Social and physical environmental factors**	OR	95% CI	*p*	OR	95% CI	*p*
**Physical environment**						
**Intercept**	1.26	[1.17–1.36]	<0.001	0.68	[0.62–0.74]	<0.001
Large negative deviation	1.00			1.00		
Negative deviation	0.75	[0.67–0.83]	<0.001	0.88	[0.78–1.00]	0.048
Small negative deviation	0.60	[0.54–0.66]	<0.001	0.85	[0.76–0.96]	0.008
Average	0.06	[0.06–0.07]	<0.001	0.18	[0.15–0.20]	<0.001
**Nuisance and insecurity**						
**Intercept**	3.21	[2.93–3.51]	<0.001	1.81	[1.64–2.00]	<0.001
Large negative deviation	1.00			1.00		
Negative deviation	0.43	[0.38–0.49]	<0.001	0.45	[0.39–0.51]	<0.001
Average	0.08	[0.07–0.09]	<0.001	0.17	[0.15–0.19]	<0.001
Small positive deviation/Positive deviation	0.02	[0.01–0.02]	<0.001	0.04	[0.03–0.05]	<0.001
**Social cohesion**						
**Intercept**	2.74	[2.52–2.97]	<0.001	1.51	[1.38–1.65]	<0.001
Large negative deviation	1.00			1.00		
Negative deviation	0.35	[0.31–0.39]	<0.001	0.43	[0.39–0.49]	<0.001
Small negative deviation	0.08	[0.07–0.09]	<0.001	0.18	[0.15–0.20]	<0.001
Average	0.03	[0.02–0.03]	<0.001	0.05	[0.05–0.06]	<0.001
**Facilities**						
**Intercept**	0.34	[0.32–0.37]	<0.001	0.25	[0.23–0.28]	<0.001
Small negative deviation/Average	1.00			1.00		
Small positive deviation	1.84	[1.64–2.06]	<0.001	1.74	[1.54–1.98]	<0.001
Positive deviation	2.09	[1.88–2.33]	<0.001	2.11	[1.87–2.38]	<0.001
Large positive deviation	2.24	[2.00–2.51]	<0.001	1.75	[1.54–1.99]	<0.001
**Housing**						
**Intercept**	2.75	[2.53–3.00]	<0.001	1.38	[1.25–1.52]	<0.001
Large negative deviation	1.00			1.00		
Negative deviation	0.54	[0.48–0.61]	<0.001	0.74	[0.65–0.84]	<0.001
Small negative deviation	0.09	[0.08–0.10]	<0.001	0.19	[0.16–0.21]	<0.001
Average	0.02	[0.02–0.03]	<0.001	0.07	[0.06–0.08]	<0.001

Odds ratio (OR). Confidence interval (CI). The definitions and details of the variables are provided in Table A2. The distribution of the defined social and physical environmental factors in 2018 for the latent classes and the total study population of children aged 0–12 years are pro-vided in Table A5.

**Table 3:** Multinomial logistic regression results of all health outcomes in 2018 and 2019 for the latent classes (reference category: first subcategory for each variable on child health outcomes).

Child Health Outcomes	OR	95% CI	*p*	OR	95% CI	*p*	OR	95% CI	*p*
**Number of health conditions** (ref = no condition)	**One condition**			**Multiple conditions**					
**Intercept**	0.57	[0.54–0.60]	<0.001	0.28	[0.26–0.30]	<0.001			
Class 1	1.00			1.00					
Class 2	1.25	[1.15–1.36]	<0.001	1.23	[1.11–1.35]	<0.001			
Class 3	1.23	[1.11–1.35]	<0.001	1.51	[1.35–1.70]	<0.001			
**Physical health condition** (ref = no)		**Yes**							
**Intercept**	0.50	[0.48–0.53]	<0.001						
Class 1	1.00								
Class 2	1.21	[1.12–1.31]	<0.001						
Class 3	1.18	[1.08–1.29]	<0.001						
**Mental health condition** (ref = no)		**Yes**							
**Intercept**	0.10	[0.09–0.11]	<0.001						
Class 1	1.00								
Class 2	0.79	[0.69–0.91]	<0.001						
Class 3	1.37	[1.19–1.57]	<0.001						
**Somatic health condition** (ref = no)		**Yes**							
**Intercept**	0.18	[0.16–0.19]	<0.001						
Class 1	1.00								
Class 2	1.40	[1.27–1.54]	<0.001						
Class 3	1.36	[1.22–1.52]	<0.001						
**Healthcare expenditure** (ref = ≥0 ≤560)	**>€560 ≤ €950**			**>€950 ≤ €1772**			**>€1772**		
**Intercept**	0.85	[0.80–0.90]	<0.001	0.73	[0.69–0.78]	<0.001	0.66	[0.62–0.71]	<0.001
Class 1	1.00			1.00			1.00		
Class 2	1.57	[1.40–1.75]	<0.001	2.16	[1.93–2.41]	<0.001	2.61	[2.34–2.92]	<0.001
Class 3	1.35	[1.19–1.52]	<0.001	1.74	[1.54–1.97]	<0.001	2.05	[1.81–2.32]	<0.001
**GP visits** (ref = 0 visits)	**1–3 visits**			**4–6 visits**			**7 or more visits**		
**Intercept**	0.66	[0.63–0.70]	<0.001	0.36	[0.34–0.39]	<0.001	0.31	[0.29–0.34]	<0.001
Class 1	1.00			1.00			1.00		
Class 2	1.42	[1.29–1.56]	<0.001	1.66	[1.49–1.85]	<0.001	1.91	[1.71–2.14]	<0.001
Class 3	1.31	[1.18–1.46]	<0.001	1.49	[1.31–1.69]	<0.001	2.01	[1.77–2.27]	<0.001
**In contact with youth care** (ref = no)	**Yes**								
**Intercept**	0.14	[0.13–0.15]	<0.001						
Class 1	1.00								
Class 2	1.06	[0.95–1.19]	0.296						
Class 3	3.05	[2.74–3.38]	<0.001						

The definitions and details of the variables are provided in Table A3. A chi-squared test was performed on the categorical variables. The distribution of the defined health outcomes in 2018 and 2019 for the latent classes and the total study population of children aged 0–12 years are provided in Table A6.

## Data Availability

The data used in this study cannot be publicly shared, as access is restricted. The data were available only under license, and accessibility is subject to the permission of ELAN and SSD.

## Abbreviations

The following abbreviations are used in this manuscript:

SES	Socioeconomic status
LCA	Latent class analysis
ACEs	Adverse childhood experiences
GP	General practitioner
ELAN	Extramural LUMC Academic Network

## Appendix A

**Table A1:** Overview of child and family demographics.

Child and Family Demographics	Definition	Categories	Data Source *
**Individual dimension**			
**Age**	Categorical age of the child in years, calculated on 1 January 2018.	0–4 years old 5–8 years old 9–12 years old	SSD
**Gender**	Gender of the child as stated at birth.	Male Female	SSD
**Country of origin**	Based on the child’s birthplace and the birthplaces of the child’s parents. An individual is categorized as ‘Dutch’ if both the person and their parents were born in the Netherlands. If the mother’s country of birth differs from the Netherlands, this country determines the classification. If the father was born in a country other than the Netherlands but the mother was born in the Netherlands, then the father’s country of birth determines the classification. If both parents were born outside the Netherlands, the mother’s country of birth is used as the determining factor. Categorized based on SSD and adjusted to frequent countries of origin in the Netherlands to fit the study population [1]. Based on the historical background of Dutch colonization, we combined Surinam, Indonesia, and the Dutch-Caribbean.	The Netherlands Europe (excluding the Netherlands) Turkey/Morocco Surinam/Indonesia/Dutch-Caribbean Other (Africa/Asia/America/Oceania)	SSD
**Social problems**	Social problems as registered by the GP in 2018 and 2019. Including problems with food/water, medical issues, with police or justice, due to violence, with family, with work/education, with housing, with partner/relation, with finances, and social problems. Included ICPC codes: Z04, Z04.01, Z04.02, Z04.03, Z04.04, Z04.05, Z24, Z27, Z28, Z29 Z29.01, Z29.02, Z29.03 Z16, Z16.01, Z16.02, Z16.03, Z18, Z19, Z20, Z21, Z21.01, Z21.02, Z22, Z23 Z12, Z12.01, Z12.02, Z13, Z13.01, Z13.02, Z13.03, Z14, Z15	No Yes	ELAN
**Household dimension**			
**Family situation**	One or both parents have been divorced in 2015–2019, or there was a shift from a two-parent household to a single-parent household. In 2018 it was registered as a single-parent household. If the registered household type includes two adults with children, an individual is considered as living in a two-parent household. This does not have to include one’s biological parents or married adults. The registered household composition refers to the number of persons registered at a home address at the same time in 2018.	Divorced parents over the past 5 years Single-parent household Household composition (*n* > 3)	SSD
**Household income**	Categorized household income based on percentage groups of standardized disposable income for private households at the start of 2018; <10th percentile is classified as low, 10th–90th percentile is classified as moderate, and >90th percentile is classified as high.	Low Moderate High	SSD
**Home ownership**	Home ownership of the home one lives in, in 2018.	Owner-occupied house Rental property without rent allowance Rental property with rent allowance	SSD
**Socioeconomic Status (SES)** **neighborhood**	Mean neighborhood SES of the neighborhood one lives in, measured by the SES-WOA score 2019 [63].	Low Middle High	SSD
**Parental dimension ****			
**Parental educational level**	Highest registered educational level achieved in 2018 by either the mother or father, according to the International Standard Classification of Education. Low included primary school and junior high school. Middle included senior high school and MBO. High included HBO and WO.	Low Middle High Missing	SSD
**Parental health**	Health condition as registered by the GP in 2016–2019. Physical health conditions: diabetes, cancer, heart failure, coronary heart disease, stroke, rheumatism, migraine, chronic obstructive pulmonary disease (COPD), asthma, dementia, hypertensive heart disease, colitis ulcerosa, (head) trauma, thyroid disorder, stomach ulcer, sexually transmitted infections/HIV, eczema, osteoporosis, miscarriage, abortion, osteoarthritis, parkinson’s disease, epilepsy, chronic neck and back pain. Included ICPC-codes: T90, T90.01, T90.02, A79, B72, B72.01, B72.02, B73, D74, D75, D76, D77, D77.01, D77.02, D77.03, D77.04, L71, L71.01, L71.02, N74, R84, R85, S77, S77.01, S77.02, S77.03, S77.04, T71, U75, U76, U77, W72, X75, X76, X76.01, X77, X77.01, X77.02, Y77, Y78, Y78.01, Y78.02, Y78.03, K78, k79, K79.01, K79.02, K80, K80.01, K80.02, K80.03, K74, K74.01, K74.02, K75, K76, K76.01, K76.02, K89, K90, K90.01, K90.02, K90.03, K86, K87, L88, L88.01, L88.02, N89, R91, R91.01, R91.02, R95, R96, R96.02, P70, P70.01, P70.02, D93,D94, D94.01, D94.01, D85, D86, D86.01, D88-D91, D91.01, D91.02, D91.03, D98, D98.01, D98.02, D98.03, A80, A81, A82, N79, N80, N80.01, N80.02, N80.03, N80.04, N81, L76, L76.01, L76.02, L76.03, L76.04, L76.05 , L76.06, L76.07, L76.08, T81, T85, T86, X70, X71, X73, X74, X74.01, X90, X91, Y70-Y72, Y76, B90, B90.01, B90.02, S86, S86.01, S86.02, S88.01, S88.02, S88.03, S88.04, S87, S88, S89, S90, S91, W80, W82, W83, N87, N87.01, L89, L90, L91, N88, L83, L83.01, L84, L84.01, L84.02, L86, L86.01 Mental health conditions: suicide attempts, burnout/stress, mood disorder, anxiety disorder, substance abuse, personality disorder, psychotic disorder, attention-deficit hyperactivity disorder (ADHD)/behavioral disorder, autism spectrum disorder (ASD), eating disorder. Included ICPC-codes: P77, P77.01, P77.02, P78, P02, P06, P06.01, P07, P03, P73, P73.02, P76, P76.01, P76.02, P01, P02.01, P74, P74.01, P74.02, P79, P79.01, P79.02, P15, P15.01, P15.02, P15.03, P15.05, P15.06, P16, P17, P18, P19, P19.01, P19.02, P80, P80.01, P80.02, P72, P98, P21, P22, P23, P04, P99, P99.01, P99.02, T06, T06.01, T06.02 Somatic health condition: headache, generalized fatigue/pain, irritable bowel syndrome (IBS), abdominal pain, neck and back Pain. Included ICPC codes: D01, D02, D04, D06, Y02, N01, N02, N90, L01, L02, L03, D93, A01, A04, A04.01	No parent physical condition One parent’s physical condition Both parents’ physical condition No parent mental condition One parent’s mental condition Both parents’ mental condition No parent somatic condition One parent’s somatic condition Both parents’ somatic condition	ELAN
**Parental social problems**	One or both parents have social problems as registered by the GP in 2016–2019. Including problems with food/water, medical issues, with police or justice, due to violence, with family, with work/education, with housing, with partner/relation, with finances and social problems. Included ICPC codes: Z04, Z04.01, Z04.02, Z04.03, Z04.04, Z04.05, Z24, Z27, Z28, Z29 Z29.01, Z29.02, Z29.03 Z16, Z16.01, Z16.02, Z16.03, Z18, Z19, Z20, Z21, Z21.01, Z21.02, Z22, Z23 Z12, Z12.01, Z12.02, Z13, Z13.01, Z13.02, Z13.03, Z14, Z15 One or both parents were registered as a victim of any criminal act (e.g., theft of belongings to sexual assault) reported to the police in 2015–2019. One or both parents were detained or suspected of a crime in 2015–2019. Based on registration of debt restructuring and/or delayed health insurance payments for more than six months for one or both parents in 2015–2019.	Parent(s) have social problems Parent(s) being victims of a crime Parent(s) being detained or suspected of a crime Parent(s) having debts	ELAN SSD SSD SSD

* data source Extramural LUMC Academic Network (ELAN) consists of registered primary care records of general practices. ELAN data was linked to routinely collected societal data (SSD) from Statistics Netherlands (SN) [23]. ** parental information from SSD and ELAN was linked through a unique parent–child identification code.

**Table A2:** Overview of social and physical environmental factors in 2018.

Social and Physical Environmental Factors	Definition	Categories *	Data Source **
Physical environment	Includes proximity to highway, main road, railroad, high voltage, transmission tower, wind turbines, green environment, dunes, open nature, water, agricultural, shops, industry, offices, semi built up, and earthquake risk, heat stress, noise pollution, flood risk, air quality, accidents, car density, and shop vacancy.	Large negative deviation Negative deviation Small negative deviation Average	Livability Index
Nuisance and insecurity	Includes violent crimes, destruction, disturbances, and nuisance and insecurity.	Large negative deviation Negative deviation Average Small positive deviation/Positive deviation	Livability Index
Social cohesion	Includes diversity of life stages, population density, mutation rate of persons, household development, and social cohesion.	Large negative deviation Negative deviation Small negative deviation Average	Livability Index
Facilities	Includes distance to education, catering industry, culture, shops, healthcare, train station, and facility density, and job accessibility.	Small negative deviation/Average Small positive deviation Positive deviation Large positive deviation	Livability Index
Housing	Includes surface area of homes, proximity to monuments, building height, housing vacancy, construction period, private rental, owner-occupied homes, overcrowding, and construction type.	Large negative deviation Negative deviation Small negative deviation Average	Livability Index

* if the outcome value is equal to the Dutch average, there is no deviation, which is generally considered ‘good’ or ‘more than sufficient’ in terms of livability. A score of more than zero indicates a positive deviation from the average Dutch Livability Index, while that of less than zero indicates a negative deviation [64]. We classified all variables into four categories based on the percentiles of the outcomes for the total study population and labeled them according to their deviation from the Dutch average: ≥0% ≤25%, >25% ≤50%, >50% ≤75%, and >75%. ** the Livability Index includes five social and physical environmental factors that shape the livability in neighborhoods as individual dimensions [26,30].

**Table A3:** Overview of child health outcomes.

Health Outcomes	Definition	Categories *	Data Source **
Number of Health conditions	Categorized number of health conditions as included within physical, mental, and somatic health conditions as registered by the GP in 2018–2019.	No condition One condition Multiple conditions	ELAN
Physical health condition	Had a physical health condition as registered by the GP in 2018–2019. Including: endocrine disorders (thyroid disorder, diabetes, or rheumatism), cancer, sexually transmitted infections/HIV, cardiac diseases (heart failure, coronary heart disease, or hypertensive heart disease), asthma, chronic obstructive pulmonary disease (COPD), eczema (including Psoriasis), migraine, Diseases Dutch National Immunization Program, (head) trauma, epilepsy, lower respiratory tract infection. Included ICPC-codes: T90, T90.01, T90.02, L88, L88.01, L88.02, A79, B72, B72.01, B72.02, B73, D75, D76, D77, D77.01, D77.02, D77.03, D77.04, L71, L71.01, L71.01, N74, R84, R85, S77, T71, U75, U76, U77, W72, S77.02, S77.03, S77.04, X75, X76, X76.01, X77, X77.01, X77.02, Y78.01, Y78.02, Y78.03, Y77, Y78, W82, W83, X70, X71, X73, X74, X74.01, B90.01, B90.02, X90, X91, Y70, Y71, Y72, Y76, B90, K78, K79.01, K79.02, K80.01, K80.02, K80.03, K79, K80, K74, K74.01, K74.02, K76.01, K76.02, K75, K76, K86, K87, R96, R96.01, R96.02, S86, S86.01, S86.02, S88.01, S88.02, S88.03, S88.04, S87, S88, S89, S90, S91, N89, A80, A81, A82, N79, N80, N80.01, N80.02, N80.03, N80.04, N81, N88, R78, R81, R81.01 R83, R83.01, A71, A74, D71, N70, N70.01, N70.02, N72, R71, R91, R91.01, R91.02, R95	No Yes	ELAN
Mental health condition	Had a mental health condition as registered by the GP in 2018–2019. Including: externalizing behavioral disorder (behavioral issues and attention-deficit hyperactivity disorder (ADHD), Substance abuse), internalizing behavioral disorder (mood disorder or anxiety disorder), other mental disorders (burnout, stress, personality disorder, autism spectrum disorder (ASD), or eating disorder). Included ICPC-codes: P15, P15.01, P15.02, P15.03, P15.05, P15.06, P19.01, P19.02, P16, P17, P18, P19, P21, P22, P23, P04, P03, P76, P76.01, P76.02, P73.02, P73, P74, P79, P79.01, P79.02, P74.01, P74.02, P02.01, P01, P72, P98, P80, P80.01, P80.02, P99.01,T06, T06.01, T06.02,P99.02, P99, P78, P78, P02, P06, P06.01, P07	No Yes	ELAN
Somatic health condition	Had a somatic health condition as registered by the GP in 2018–2019. Including: headache disorders, (chronic) neck and back pain, generalized fatigue/pain, overweight/obesity, gastrointestinal conditions (including irritable bowel syndrome (IBS) and abdominal pain). Included ICPC-codes: N01, N02, N90, L01, L02, L03, L83, L83.01, L84, L84.01, L84.02, L86.01, L86, A01, A04, A04.01, T82, T83, D93, D94, D94.01, D94.02, D85, D86, D86.01, D88-D91, D91.01, D91.02, D91.03, D98, D98.01, D98.02, D98.03, D01, D02, D04, D06, Y02	No Yes	ELAN
Healthcare expenditure	Total healthcare costs between 2018 and 2019. Includes all costs associated with the Health Insurance Act (e.g., GP costs and hospital care costs), excluding youth care costs.	≥0 ≤560 >€560 ≤€950 >€950 ≤€1772 >€1772 Missings	SSD
GP visits	Total number of GP visits during 2018–2019. Excluding repeat prescriptions, flu vaccines, internal consultations, mail processing, notes, and missed appointments.	0 visits 1–3 visits 4–6 visits 7 or more visits	ELAN
In contact with (inpatient or outpatient) youth care	Children to whom, during (part of) the reporting periods, one or more forms of assistance or care were provided under the Youth Act, such as support for psychological or parenting problems, or who received services and support through the Dutch Youth Protection Services. The juvenile court decides on a child protection measure to eliminate any threats to the safe development of a child, such as an unsafe family environment. This decision follows an investigation by the Council for Child Protection [4]. Included years: 2018–2019.	No Yes	SSD

* the GP visits in 2018 and 2019 for the selected regions were classified into four categories according to the percentiles of the outcomes for the total study population: ≥0% ≤25%, >25% ≤50%, >50% ≤75%, and >75%. The costs were classified into four categories based on the percentiles of the outcomes for the total study population: ≥0% ≤25%, >25% ≤50%, >50% ≤75%, and >75%. ** data source Extramural LUMC Academic Network (ELAN) consists of registered primary care records of general practices. ELAN data was linked to routinely collected societal data (SSD) from Statistics Netherlands (SN) [23].

**Table A4:** Fit statistics of the latent class analysis models *.

Number of Class	Akaike Information Criterion (AIC)	Bayesian Information Criterion (BIC)	G-Square	Chi-Square Goodness of Fit	Residual Degrees of Freedom	Log Likelihood	Entropy
**1**	384,962.3	385,182.3	115,321.10	1,315,665,127	14,518	−192,452.20	-
**2**	363,567.3	364,014.8	94,622.32	380,063,330	14,488	−181,724.60	0.80
**3**	**357,324.1**	**357,999.2**	**88,696.10**	**419,886,409**	**14,458**	**−178,573.10**	**0.82**
**4**	354,395.4	355,298.1	85,886.72	115,893,466	14,428	−177,078.70	0.79
**5**	352,336.7	353,466.9	83,912.73	45,522,607	14,398	−176,019.30	0.76
**6**	350,727.8	352,085.5	82,563.98	35,120,814	14,368	−175,184.90	0.75

The model represents the number of classes included in the LCA model in bold. * LCA was performed using the ‘poLCA’ package. Model selection was guided by the Akaike and Bayesian information criteria, and latent class solutions were assessed based on entropy, with higher values reflecting clearer class separation [33]. The estimation algorithm used up to 3000 iterations and 20 repetitions with classes labeled by posterior probabilities of social factors, indicating the membership likelihood given their observed social factors [65,66].

**Table A5:** The distribution of the defined social and physical environmental factors in 2018 for the latent classes and the total study population of children aged 0–12 years.

	Class 1	Class 2	Class 3	Total Study Population
**Social and physical environmental factors**	N (%)	N (%)	N (%)	N (%)
**Physical environment**				
**Large negative deviation**	639 (8.8%)	2196 (50.9%)	1094 (36.9%)	3569 (24.5%)
**Negative deviation**	1059 (14.6%)	1356 (31.4%)	914 (30.8%)	3487 (24.0%)
**Small negative deviation**	2352 (32.4%)	625 (14.5%)	673 (22.7%)	3854 (26.5%)
**Average**	3217 (44.3%)	136 (3.2%)	286 (9.6%)	3637 (25.0%)
**Nuisance and insecurity**				
**Large negative deviation**	612 (8.4%)	1963 (45.5%)	1110 (37.4%)	3685 (25.3%)
**Negative deviation**	1127 (15.5%)	1565 (36.3%)	913 (30.8%)	3605 (24.8%)
**Average**	2337 (32.2%)	623 (14.4%)	724 (24.4%)	3684 (25.3%)
**Small positive deviation/Positive deviation**	3191 (43.9%)	162 (3.8%)	220 (7.4%)	3573 (24.6%)
**Social cohesion**				
**Large negative deviation**	789 (10.9%)	2159 (50.1%)	1192 (40.2%)	4140 (28.5%)
**Negative deviation**	1712 (23.6%)	1622 (37.6%)	1121 (37.8%)	4455 (30.6%)
**Small negative deviation**	1464 (20.1%)	307 (7.1%)	386 (13.0%)	2157 (14.8%)
**Average**	3302 (45.4%)	225 (5.2%)	268 (9.0%)	3795 (26.1%)
**Facilities**				
**Small negative deviation/Average**	2227 (30.6%)	763 (17.7%)	565 (19.0%)	3555 (24.4%)
**Small positive deviation**	1650 (22.7%)	1041 (24.1%)	730 (24.6%)	3421 (23.5%)
**Positive deviation**	1847 (25.4%)	1325 (30.7%)	988 (33.3%)	4160 (28.6%)
**Large positive deviation**	1543 (21.2%)	1184 (27.5%)	684 (23.1%)	3411 (23.4%)
**Housing**				
**Large negative deviation**	695 (9.6%)	1914 (44.4%)	958 (32.3%)	3567 (24.5%)
**Negative deviation**	1096 (15.1%)	1630 (37.8%)	1120 (37.7%)	3846 (26.4%)
**Small negative deviation**	2315 (31.9%)	573 (13.3%)	595 (20.1%)	3483 (23.9%)
**Average**	3161 (43.5%)	196 (4.5%)	294 (9.9%)	3651 (25.1%)

The definitions and details of the variables are provided in Table A2.

**Table A6:** The distribution of all health outcomes in 2018 and 2019 for the latent classes and the total study population of children aged 0–12 years.

	Class 1	Class 2	Class 3	Total Study Population
**Child health outcomes**	N (%)	N (%)	N (%)	N (%)
**Number of health conditions**				
**No condition**	3928 (54.1%)	2082 (48.3%)	1397 (47.1%)	7407 (50.9%)
**One condition**	2234 (30.7%)	1481 (34.3%)	975 (32.9%)	4690 (32.2%)
**Multiple conditions**	1105 (15.2%)	975 (32.9%)	595 (20.1%)	2450 (16.8%)
**Physical health condition**				
**No**	4843 (66.6%)	2686 (62.3%)	1865 (62.9%)	9394 (64.6%)
**Yes**	2424 (33.4%)	1627 (37.7%)	1102 (37.1%)	5153 (35.4%)
**Mental health condition**				
**No**	6597 (90.8%)	3991 (92.5%)	2605 (87.8%)	13,193 (90.7%)
**Yes**	670 (9.2%)	322 (7.5%)	362 (12.2%)	1354 (9.3%)
**Somatic health condition**				
**No**	6182 (85.1%)	3463 (80.3%)	2395 (80.7%)	12,040 (82.8%)
**Yes**	1085 (14.9%)	850 (19.7%)	572 (19.3%)	2507 (17.2%)
**Healthcare expenditure**				
**≥0 ≤560**	2230 (30.7%)	763 (17.7%)	621 (20.9%)	3614 (24.8%)
**>€560 ≤€950**	1891 (26.0%)	1013 (23.5%)	709 (23.9%)	3613 (24.8%)
**>€950 ≤€1772**	1635 (22.5%)	1208 (28.0%)	793 (26.7%)	3636 (25.0%)
**>€1772**	1473 (20.3%)	1316 (30.5%)	840 (28.3%)	3629 (24.9%)
**Missings**	38 (0.5%)	13 (0.3%)	4 (0.1%)	55 (0.4%)
**GP visits**				
**0 visits**	3114 (42.9%)	1376 (31.9%)	979 (33.0%)	5469 (37.6%)
**1–3 visits**	2059 (28.3%)	1293 (30.0%)	849 (28.6%)	4201 (28.9%)
**4–6 visits**	1120 (15.4%)	822 (19.1%)	525 (17.7%)	2467 (17.0%)
**7 or more visits**	974 (13.4%)	822 (19.1%)	614 (20.7%)	2410 (16.6%)
**In contact with youth care**				
**No**	6357 (87.5%)	3744 (86.8%)	2066 (69.6%)	12,167 (83.6%)
**Yes**	910 (12.5%)	569 (13.2%)	901 (30.4%)	2380 (16.4%)

The definitions and details of the variables are provided in Table A3.
